# Engineering Atomically Precise Cu_4_I_4_ Nanoclusters With Integrated Adsorption and Hydrogenation Functions for Efficient Photocatalytic Nitrate‐to‐Ammonia Conversion

**DOI:** 10.1002/anie.5529008

**Published:** 2026-08-29

**Authors:** Wanting Zhang, Yuehua Li, Junjie Jin, Chunwei Dong, Xi Cao, Xingchi Li, Xuanjun Wu, Jiaqi Wang, Atta Ullah, Gaspar Ferreyra Vaggione, Rodrigo A. Iglesias, Yun Chen, Emiliano Cortés, Dengsong Zhang

**Affiliations:** ^1^ International Joint Laboratory of Catalytic Chemistry Innovation Institute of Carbon Neutrality Department of Chemistry College of Sciences Shanghai University Shanghai China; ^2^ Nanoinstitute Munich, Faculty of Physics Ludwig‐Maximilians‐Universität (LMU) Munich Germany; ^3^ School of Physics and Electronic Information Engineering Guangxi Minzu Normal University Chongzuo Guangxi China; ^4^ Department of Physical Chemistry School of Chemistry Tel Aviv University Tel Aviv Israel; ^5^ INFIQC CONICET, Departamento de Fisicoquímica Facultad de Ciencias Químicas Universidad Nacional de Córdoba (UNC) Córdoba Argentina

**Keywords:** ammonia synthesis, catalytic interface, nanocluster, photocatalysis, photocatalytic nitrate reduction

## Abstract

Photocatalytic nitrate reduction to ammonia offers a sustainable route that couples environmental remediation with nitrogen resource utilization. However, this transformation suffers from sluggish kinetics and insufficient hydrogenation, leading to poor selectivity and efficiency. Herein, we report the deposition of Cu_4_I_4_ nanocluster onto TiO_2_ (Cu_4_I_4_/TiO_2_) that achieves efficient and selective photocatalytic nitrate reduction to ammonia under mild conditions. The active site derived from an atomically precise Cu_4_I_4_Py_4_ (Py = pyridine) nanocluster introduces sterically accessible copper sites with intrinsically unsaturated coordination and enhanced Lewis acidity for nitrate adsorption and activation. The iodide ligands in Cu_4_I_4_ motif directly bonded to copper atoms are suggestive of promoting the generation of reactive hydrogen species (*H). The atomic‐scale proximity of copper and iodide sites facilitates efficient *H utilization in the multistep hydrogenation toward NH_3_. Consequently, Cu_4_I_4_/TiO_2_ delivers an ammonia generation rate of 26.8 mmol·g_cat_^−1^·h^−1^ with good selectivity in a sacrificial‐reagent‐assisted photocatalytic nitrate reduction system. In situ characterizations and theoretical calculations support the plausible cooperative dual‐site mechanism, which synergistically ensures the deep hydrogenation of nitrate and its intermediates to ammonia. This work establishes an atomic‐level design paradigm for constructing multifunctional nanocluster catalysts that address the selectivity and efficiency challenges inherent to complex multiple proton/electron‐involved reactions.

## Graphical abstract

Efficient photocatalytic nitrate‐to‐ammonia conversion is achieved over an atomically precise Cu_4_I_4_ motif through dual‐site cooperation. Lewis‐acidic Cu centers promote nitrate adsorption and activation, while adjacent I sites facilitate active hydrogen generation for stepwise hydrogenation. This synergistic mechanism boosts ammonia yield and selectivity, underscoring the structural advantage of well‐defined Cu_4_I_4_ sites for selective nitrate reduction.

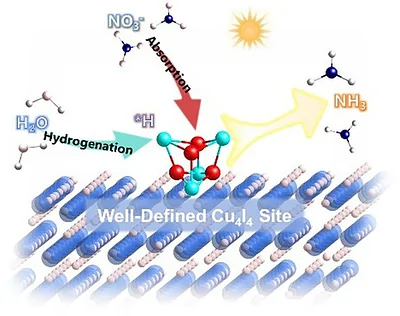

## 1 Introduction

Ammonia (NH_3_) serves as an essential feedstock with critical roles in fertilizer production, chemical synthesis, and emerging carbon‐free energy applications [1, 2]. Currently, global ammonia production is dominated by the Haber–Bosch process, which operates under harsh conditions (350°C–450°C, 150 to 200 bar) and is responsible for 1%–2% of global energy consumption and ∼1.5% of carbon dioxide emissions [3, 4]. In the pursuit of a green and sustainable society, solar‐driven ammonia synthesis has emerged as a promising renewable alternative to the Haber–Bosch process [5]. However, direct nitrogen photoreduction is severely constrained by the formidable strength of the N≡N triple bond (∼941 kJ•mol^−1^). By contrast, nitrate (NO_3_^−^), characterized by a substantially weaker bond dissociation energy (∼204 kJ•mol^−1^), has emerged as an accessible and feasible nitrogen source for ammonia photosynthesis under ambient conditions [6]. Beyond its thermodynamic advantages, nitrate represents a ubiquitous environmental contaminant, posing significant risks to ecosystem and public health [7]. Accordingly, photocatalytic nitrate reduction to ammonia confers dual benefits: establishing a sustainable route for ammonia production while concurrently contributing to the remediation of the aquatic environment.

Despite its promise, photocatalytic nitrate reduction to ammonia still faces inherent challenges, including competing multi‐electron reduction pathways that yield a broad distribution of products (e.g., nitrogen and nitrite), and low quantum efficiencies [8]. The key challenge lies in precisely engineering the interfacial active sites that can effectively harness photogenerated electrons to selectively drive ammonia generation, while simultaneously suppressing parasitic pathways such as the hydrogen evolution reaction and reductive coupling to dinitrogen [9]. The overall photocatalytic performance is primarily dictated by two factors: efficient nitrate adsorption and activation to initiate the photocatalytic reduction, and sufficient atomic hydrogen (*H) supply to enable complete hydrogenation [10, 11]. Specifically, the photocatalytic reduction of nitrate‐to‐ammonia involves multiple proton/electron‐coupled steps, yet the sluggish kinetics of *H generation and migration from the interfacial water dissociation substantially impede the overall reaction efficiency [12]. Thus, achieving high selectivity and activity toward ammonia generation necessitates not only stabilization of key nitrogen intermediates but also acceleration of interfacial water adsorption–dissociation to generate ample *H for subsequent hydrogenation steps. To date, a wide range of semiconductor and plasmonic materials have been investigated for solar‐driven ammonia synthesis [13]. Plasmonic systems are attractive because localized surface plasmon resonance enables strong light confinement and hot‐carrier generation; however, they are often limited by rapid charge recombination. Conventional semiconductor photocatalysts likewise suffer from wide bandgaps and sluggish surface redox kinetics. Although photoelectrocatalytic strategies employing an external bias can enhance charge separation, the applied potential may induce structural distortion or leaching of active species, thereby compromising atomic‐level control [14]. Therefore, the development of purely photocatalytic systems operating under mild, bias‐free conditions remains highly desirable to ensure both sustainability and structural integrity of well‐defined catalytic sites, thereby offering a promising strategy to address the intrinsic challenges associated with nitrate‐to‐ammonia conversion.

Recently, atomically precise nanoclusters have emerged as a distinct class of catalytic materials. Owing to the diversity and unparalleled design versatility, nanoclusters hold immense promise for enabling innovative chemical transformations [15]. Their well‐defined atomic arrangements, discrete electronic states, and quantum size effects bridge the gap between molecular catalysts and nanoparticles, enabling direct and precise correlation between structure and catalytic function [16]. When integrated with suitable substrates, nanoclusters generate active sites with precisely tailored geometric and electronic configurations, often outperforming single‐atom and nanoparticle catalysts due to their unique adsorption characteristics and multi‐electron transfer capabilities [17]. Crucially, their compositional and structural tunability, such as controlled variation of atomicity, configuration, and alloying, provides powerful opportunities to optimize light harvesting, charge separation, and active‐site chemistry for selective ammonia production [18]. By combining structural precision, high catalytic activity, and mechanistic clarity, constructing nanocluster catalysts represents a promising strategy to overcome current limitations and advance photocatalytic ammonia synthesis toward efficient and sustainable application.

Copper‐based composite photocatalysts have received considerable attention for photocatalytic ammonia synthesis owing to their 3d^10^ electronic configuration, which enables strong back‐donation interactions and facilitates the adsorption and activation [19]. For example, Cu_2_O sub‐nanoclusters that are on‐site constructed have been demonstrated to serve as effective active sites for photocatalytic nitrate‐to‐ammonia conversion [20]. However, such strategies based on the aggregation of metal precursors on the support often lead to random atomicity and poorly defined structures of the nanoclusters. Moreover, their performance remains fundamentally constrained by the insufficient supply of reactive hydrogen species, underscoring the need to introduce complementary sites that enhance the hydrogenation process [21]. To address this issue, a synergistic Ag‐Cu dual‐atom catalyst has been proposed, where the Cu_1_ and Ag_1_ dual sites are responsible for the nitrate activation and hydrogenation, respectively, thereby ensuring the deep hydrogenation of nitrate [4]. However, the interatomic distance between Ag–Cu dual sites critically influences the transfer of intermediates and protons, and excessive separation will lead to sluggish reaction kinetics and reduced selectivity. In this regard, the structural versatility of atomic copper‐based nanoclusters provides a valuable platform for constructing nanocluster‐based photocatalysts, in which nitrate adsorption and hydrogenation sites can be integrated within a single nanocluster in proximity. Herein, we propose an atomic‐level design of a Cu_4_I_4_/TiO_2_ photocatalyst featuring highly exposed low‐coordinated copper sites adjacent to iodide atoms, which enables efficient photoreduction of nitrate to ammonia with formic acid as a sacrificial reagent. The photocatalyst is derived from an atomically precise Cu_4_I_4_Py_4_ (Py = pyridine) nanocluster, where the labile pyridine ligands can be removed under mild thermal treatment, leaving the cubane Cu_4_I_4_ core anchored to TiO_2_ via Cu–O interactions. The strong Cu–O interfacial coupling ensures uniform nanocluster dispersion and generates low‐coordinated Cu sites with strong Lewis acidity that enhance nitrate adsorption and activation. Meanwhile, iodide atoms facilitate interfacial water activation, providing abundant reactive hydrogen for the hydrogenation process. These synergistic effects enable Cu_4_I_4_/TiO_2_ to achieve outstanding ammonia yield and selectivity.

## 2 Results and Discussion

Tetranuclear cubane copper(I) halide nanoclusters with the general formula [Cu_4_I_4_L_4_] (L = N─ or P─ donor ligands) are well known for their thermochromic photoluminescence properties. The [Cu_4_I_4_L_4_] nanocluster consists of four copper atoms and four iodide atoms arranged alternately at the eight corners of a cubane‐like [Cu_4_I_4_] core (Figure S1) [22]. Notably, the cubane‐type structural motif is the most representative configuration in the nanocluster family across a wide range of compositions [23, 24]. The stability and robustness of the cubane‐type Cu_4_I_4_ motif highlight its potential to preserve structural integrity after immobilization on a support. In the [Cu_4_I_4_L_4_] nanocluster, each copper atom is further coordinated to an organic ligand with an N or P atom. Thus, the copper atoms adopt a four‐coordinate tetrahedral configuration with three iodide atoms and one ligand (Figure S2). The Cu─I bond is generally stronger than the Cu─L coordination bond, particularly in case of Cu–N interactions [22]. This dual coordination environment, combining robust Cu─I bonds with labile Cu─N linkages, offers structural flexibility for the rational design of functional active sites. Moreover, copper (I) iodide exhibits a low solubility product constant (*K_sp_* = 1.27 × 10^−12^), which is several orders of magnitude lower than those of known sparingly soluble salts such as calcium carbonate (*K_sp_* = 3.36 × 10^−9^) and barium sulfate (*K_sp_* = 1.08 × 10^−10^). This low solubility highlights the intrinsic stability of Cu─I species, supporting the robustness and long‐term reliability as catalytically active centers under aqueous reaction conditions. In this context, Cu_4_I_4_Py_4_ nanocluster was selected as a model precursor to construct a composite photocatalyst for efficient nitrate reduction to ammonia. First, Cu_4_I_4_Py_4_ was synthesized according to the previous procedure [25], and its successful formation was confirmed by x‐ray diffraction (XRD) pattern and Raman spectrum (Figures S3 and S4). Under ambient conditions, the as‐synthesized Cu_4_I_4_Py_4_ exhibits intense yellow luminescence upon UV light irradiation (Figure S5). A broad emission band centered at 575 nm is observed due to the “cluster‐centered” low energy transition, consistent with the well‐defined cubane [Cu_4_I_4_L_4_] nanoclusters [26].

The reasonable solubility of [Cu_4_I_4_L_4_] in diverse solvents ensures its molecular uniformity [27], enabling the facile and homogeneous preparation of Cu_4_I_4_/TiO_2_ composites, as schematically illustrated in Figure 1a. The synthesis involves a two‐step process: impregnation of Cu_4_I_4_Py_4_ nanoclusters onto TiO_2_, followed by a calcination treatment. Owing to the molecular‐level dispersion of Cu_4_I_4_Py_4_ in solution, the nanoclusters can be uniformly anchored on the surface of TiO_2_. The subsequent calcination process is crucial: the temperature should be sufficiently high to remove the organic ligands while avoiding nanocluster aggregation into bulk particles. To determine the optimal calcination conditions, thermogravimetry analyses (TGA) of Cu_4_I_4_Py_4_ were performed. As shown in Figure 1b, the TGA curve of Cu_4_I_4_Py_4_ exhibits a substantial weight loss between 120°C–160°C, corresponding to the dissociation of pyridine ligands. The weight loss of approximately 27% agrees well with the theoretical pyridine content (29%), suggesting complete ligand removal in this temperature range. Typically, metal nanoclusters protected by thiolate and phosphine ligands require significantly high temperatures for ligand removal (>300°C) [28]. The annealing treatment at such high temperatures inevitably leads to the aggregation of nanoclusters into bulk particles. In contrast, the pyridine ligands in Cu_4_I_4_Py_4_ can be removed at a much lower temperature, avoiding the aggregation. Therefore, a calcination temperature of 140°C was selected to ensure complete removal of pyridine ligands while maintaining the structural integrity.

**FIGURE 1:**
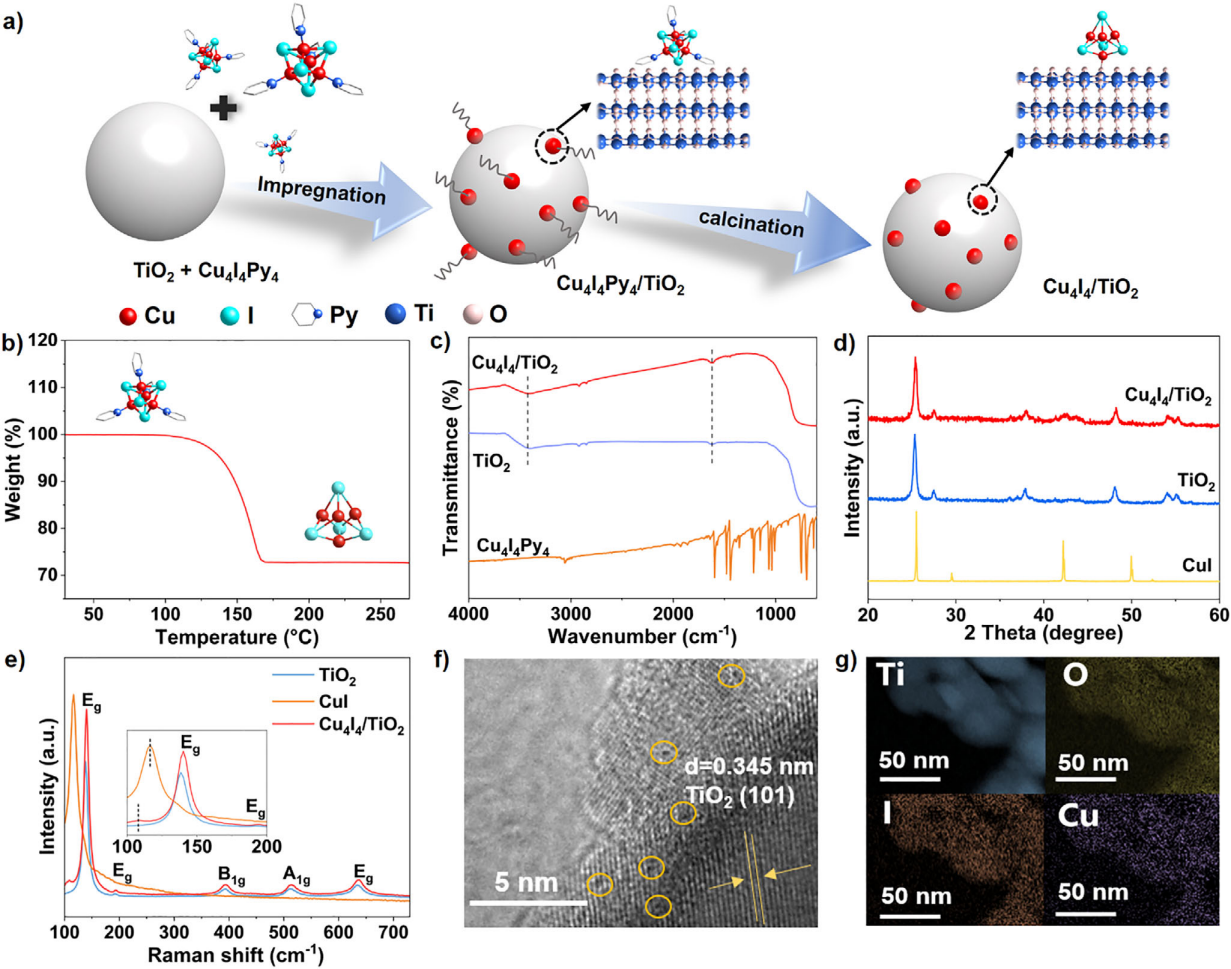
Morphology and structure characterization of Cu_4_I_4_/TiO_2_. (a) Synthesis procedure for Cu_4_I_4_/TiO_2_, (b) TGA curve of Cu_4_I_4_Py_4_, (c) FTIR spectra of Cu_4_I_4_Py_4_, TiO_2_, and Cu_4_I_4_/TiO_2_. (d) XRD patterns and (e) Raman spectra of CuI, TiO_2_, and Cu_4_I_4_/TiO_2_. (f) HR‐TEM image and (g) electron energy loss spectroscopy (EELS) mapping images of Cu_4_I_4_/TiO_2_.

Fourier transform infrared spectroscopy (FTIR) was further employed to confirm the removal of pyridine ligands. As shown in Figure 1c, pure TiO_2_ shows characteristic absorption bands at 3000–3560 and 1640 cm^−1^, corresponding to the vibration of H_2_O on the surface of TiO_2_ [29]. Cu_4_I_4_Py_4_ shows a series of sharp peaks in the range of 500–1600 cm^−1^, originating from the vibration of the C═C and C─N bonds of pyridine [30]. In contrast, these peaks are absent in Cu_4_I_4_/TiO_2_, confirming the successful removal of pyridine. XRD and Raman spectroscopy were performed to analyze the phase composition and interfacial interaction. As shown in Figures 1d and S6, Cu_4_I_4_/TiO_2_ exhibits identical XRD pattern with that of pristine TiO_2_, possessing a mixture phase of anatase and rutile (JCPDS File No. 84–1286 and 78–1508). Remarkably, no diffraction peaks corresponding to bulk CuI are observed for Cu_4_I_4_/TiO_2_, revealing that Cu_4_I_4_ nanoclusters are uniformly dispersed on the surface of TiO_2_ without aggregation. Raman spectrum of TiO_2_ exhibits characteristic peaks at 138, 395, 514, and 635 cm^−1^ (Figure 1e), which are attributed to the typical vibrations of anatase TiO_2_ [31, 32]. Upon Cu_4_I_4_ loading, a new peak located at 108 cm^−1^ appears, and the Raman peaks of TiO_2_ slightly shift toward the higher wavenumber, demonstrating the successful deposition of Cu_4_I_4_ nanocluster on TiO_2_ and the strong interaction between Cu_4_I_4_ and TiO_2_. Importantly, the absence of a peak at 117 cm^−1^, which is characteristic of CuI [33], confirms that Cu_4_I_4_ nanoclusters on TiO_2_ retain their molecular structure without aggregation into bulk CuI. In addition, the high‐resolution transmission electron microscopy (HR‐TEM) image of Cu_4_I_4_/TiO_2_ shows clear lattice fringes with a *d*‐spacing of 0.345 nm (Figures 1f and S7), which corresponds to the (101) facet of TiO_2_ [34]. The uniformly dispersed dark dots marked by yellow circles can be assigned to Cu_4_I_4_ nanoclusters. Elemental mappings (Figure 1g) further confirm that Cu and I elements are homogeneously distributed on the surface of TiO_2_. UV–vis diffuse reflectance spectroscopy (UV–vis DRS) reveals the enhanced light absorption of Cu_4_I_4_/TiO_2_ compared with pristine TiO_2_ (Figure S8), which can be attributed to the strong interaction and light‐harvesting contribution from the Cu_4_I_4_ nanocluster [35]. The copper content is determined to be 0.96% via an inductively coupled plasma optical emission spectrometer (Table S2), which is consistent with the nominal loading of the prepared 5%Cu_4_I_4_/TiO_2_. Collectively, these results demonstrate that the pyridine ligands in Cu_4_I_4_Py_4_ can be removed under mild calcination, leading to the successful and uniform dispersion of Cu_4_I_4_ nanoclusters without noticeable aggregation.

X‐ray photoelectron spectroscopy (XPS) was performed to investigate the chemical states and the electronic interaction of the as‐prepared samples. The survey spectrum of Cu_4_I_4_/TiO_2_ confirms the existence of Ti, O, Cu, and I elements (Figure S9). As shown in Figure 2a, the Cu 2p_1/2_ and Cu 2p_3/2_ peaks located at 952.4 and 932.5 eV, respectively, are the characteristics of Cu^+^ species in Cu_4_I_4_Py_4_ [36]. In the I 3d spectrum of Cu_4_I_4_Py_4_ (Figure 2b), the peaks located at 631.2 and 619.8 eV correspond to the 3d_3/2_ and 3d_5/2_ of I^−^, respectively [37]. For pristine TiO_2_, the Ti 2p spectrum (Figure 2c) displays two peaks at 458.4 and 464.1 eV, assigned to the Ti^4+^ 2p_3/2_ and 2p_1/2_, while the O 1s spectrum (Figure 2d) exhibits two components centered at 529.5 and 531.2 eV, corresponding to lattice oxygen and surface oxygen species [38]. After the incorporation of Cu_4_I_4_ onto TiO_2_, all constituent elements exhibit comparable oxidation state but with discernible binding energy shifts. Notably, the Cu 2p and I 3d peaks of Cu_4_I_4_/TiO_2_ shift toward lower binding energy than those of Cu_4_I_4_Py_4_ and CuI (Figure S10). Specifically, relative to Cu_4_I_4_Py_4_ nanocluster, the Cu 2p and I 3d peaks in Cu_4_I_4_/TiO_2_ shift toward lower binding energies by 0.6 and 1.1 eV, respectively, indicating the increased electron density at Cu_4_I_4_ sites. In contrast, relative to TiO_2_, the O 1s and Ti 2p peaks of Cu_4_I_4_/TiO_2_ shift toward higher binding energy by 0.3 and 0.1 eV, respectively, indicative of electron transfer from TiO_2_ to Cu_4_I_4_. The observed XPS binding energy shifts reflect the strong electronic interaction and high dispersion of Cu_4_I_4_ on the TiO_2_ support. Considering that pyridine acts as an electron‐donating ligand, its removal leads to an electron‐deficient Cu environment, which is subsequently compensated by electron donation from the TiO_2_ substrate through strong interfacial Cu–O interactions.

**FIGURE 2:**
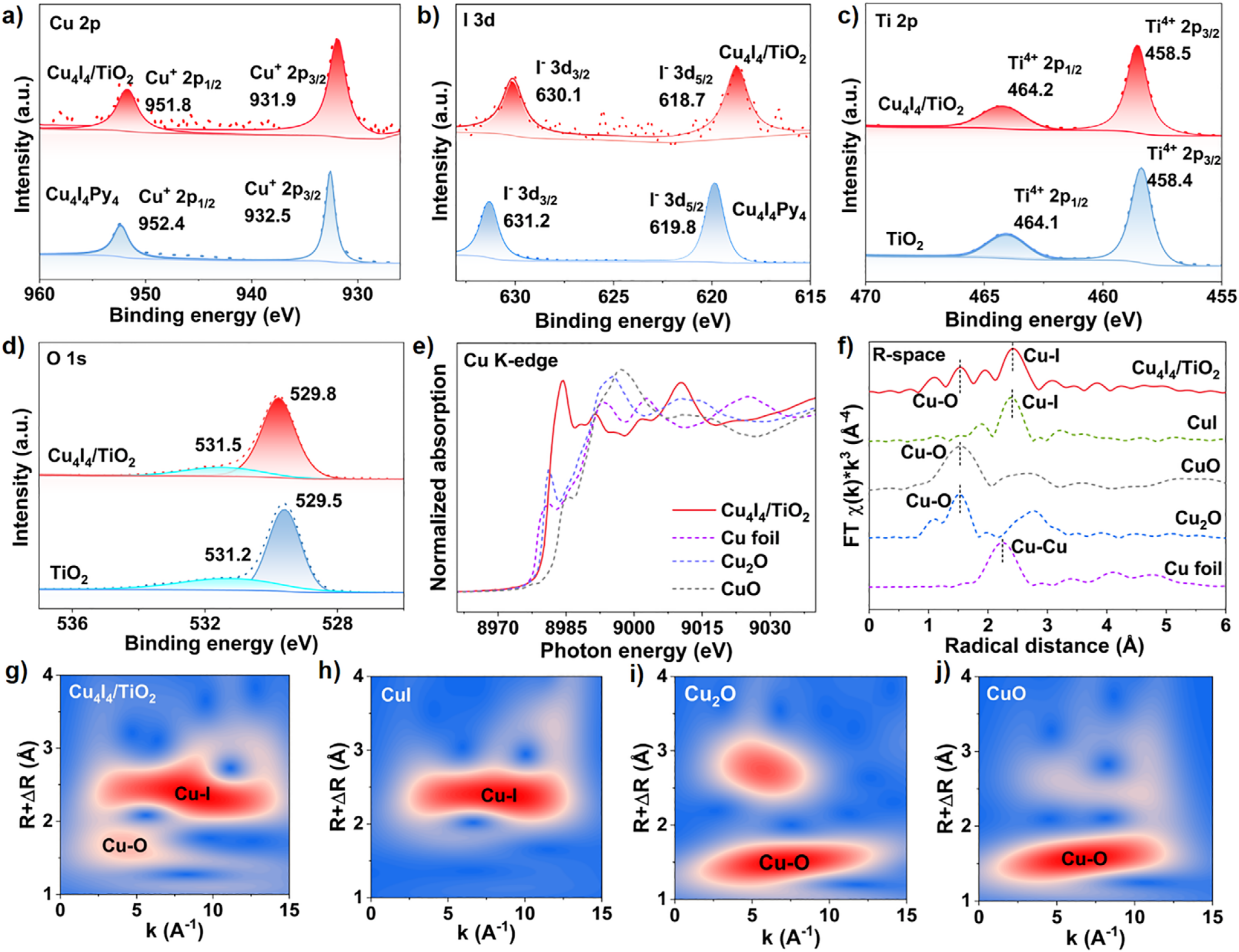
Structural characterization and local environment of Cu_4_I_4_/TiO_2_. (a) Cu 2p, (b) I 3d, (c) Ti 2p, and (d) O 1s XPS spectra of the catalysts. (e) Cu *K*‐edge XANES spectra of Cu foil, Cu_2_O, CuO, and Cu_4_I_4_/TiO_2_; (f) the FT‐EXAFS spectra of Cu foil, Cu_2_O, CuO, CuI, and Cu_4_I_4_/TiO_2_. WT contour plots of Cu *K*‐edge EXAFS signals of (g) Cu_4_I_4_/TiO_2_, (h) CuI, (i) Cu_2_O, and (j) CuO.

To further elucidate the local coordination environment of Cu species, x‐ray absorption spectroscopy (XAS) was carried out. As depicted in Figure 2e, the Cu *K*­edge x‐ray absorption near‐edge structure (XANES) spectrum of Cu_4_I_4_/TiO_2_ exhibits a nearly identical absorption edge to that of Cu_2_O, confirming that the Cu species predominantly exist in the +1 oxidation state, which is consistent with the XPS results [39]. The k^3^‐weighted Fourier‐transformed extended x‐ray absorption fine structure (EXAFS) oscillation is further analyzed. As shown in Figure 2f, Cu_4_I_4_/TiO_2_ exhibits two dominant peaks at 1.5 and 2.4 Å, which can be assigned to the Cu─O and Cu─I first‐shell coordination, respectively [40]. Importantly, no Cu–Cu scattering characteristic of metallic copper is detected, suggesting that the resulting nanocluster catalyst retains the key features of the Cu_4_I_4_ motif from the parent nanocluster with a well‐defined Cu–I coordination. The wavelet transforms of EXAFS spectra further support this observation, showing that the Cu bonding environment in Cu_4_I_4_/TiO_2_ lies between that of CuI and Cu_2_O (Figure 2g–j), evidencing the interfacial Cu–O interactions between the Cu_4_I_4_ species and TiO_2_ substrate. Additionally, quantitative EXAFS fitting yields an average coordination number (CN) of 3.2 for the Cu atom (Figure S11 and Table S3), comprising Cu–O and Cu–I coordination. In the parent Cu_4_I_4_Py_4_ nanocluster, the crystal structure shows that each Cu atom is coordinated by three I atoms and one pyridine N atom (CN = 4). Upon pyridine removal, partial Cu atoms form strong Cu─O bonds with TiO_2_. However, the CN (0.7) of the Cu─O bond is substantially lower than the CN (1.0) of Cu─N in the parent Cu_4_I_4_Py_4_ nanocluster, indicating the formation of low‐coordinated Cu centers in Cu_4_I_4_/TiO_2_. Given the cubane‐type geometry of the Cu_4_I_4_ motif and the corner sites of Cu atoms, these structural modifications lead to highly exposed, low‐coordinated Cu sites.

The photocatalytic performance of as‐prepared catalysts toward nitrate reduction to ammonia was evaluated under UV–vis irradiation in an Ar atmosphere, using formic acid (HCOOH) as a sacrificial reagent to accelerate the consumption of photogenerated holes. The ammonia concentration was determined using the indophenol blue spectrophotometric (IBS) method (Figure S12). The nitrate and nitrite concentrations during the reaction were determined by ion chromatography (Figures S13 and S14). The key photocatalytic experiments were repeated three times, and the corresponding data are presented with error bars. As shown in Figures 3a and S15, pristine TiO_2_ exhibits a modest ammonia yield of 0.61 mmol·g_cat_^−1^·h^−1^ after 1 h of illumination, with a low ammonia selectivity of 16.3% and a nitrate conversion of 66.4%. Upon introducing Cu_4_I_4_ nanocluster, both the ammonia yield and nitrate conversion are dramatically enhanced. In particular, 5% Cu_4_I_4_/TiO_2_ delivers an optimal ammonia yield of 8.11 mmol·g_cat_^−1^·h^−1^, which is virtually 12‐fold as high as that of bare TiO_2_ (Figure 3a). Meanwhile, the ammonia selectivity is significantly improved from 16.3% to 83.5% (Figure 3b). Crucially, ammonia and N_2_ are the only detectable nitrogen‐containing products under the present reaction conditions (Figures S16 and S17). No other gaseous or liquid‐phase nitrogen‐containing products such as NH_2_OH and N_2_H_4_ are detected (Figures S18–20). Upon extending the irradiation time to 2 h, the concentration of the intermediate nitrite decreases to zero, accompanied by a further increase in ammonia selectivity to 89.3%. Notably, an excessive loading amount of Cu_4_I_4_ nanocluster leads to decreased photocatalytic activity due to the formation of less active CuI species and thereby a reduced number of efficient nanocluster active sites (Figure S21). In addition, the calcination temperature plays a critical role in determining the catalytic performance. As shown in Figure S22, high‐temperature treatment induces the aggregation of Cu_4_I_4_ into the CuI phase, thereby diminishing the number of active sites and resulting in decreased photocatalytic efficiency. Moreover, by optimizing the reaction parameters, such as nitrate concentration, catalyst loading amount, and formic acid content, the ammonia generation rate can reach 26.8 mmol·g_cat_^−1^·h^−1^ (Figure S23), with an AQY of 1.95% at 350 nm (Table S4), positioning Cu_4_I_4_/TiO_2_ among the state‐of‐the‐art catalysts (Table S5). These results demonstrate that the integration of Cu_4_I_4_ nanocluster with TiO_2_ not only enhances activity but also fundamentally steers the photocatalytic nitrate reduction pathway from N_2_ evolution toward selective ammonia formation. Furthermore, we have performed photocatalytic hydrogen evolution experiments to evaluate the competing reaction (Figure S24). As shown in Figure S25, the Cu_4_I_4_/TiO_2_ exhibits enhanced hydrogen evolution compared with TiO_2_ under identical reaction conditions, which can be attributed to the improved charge transfer and separation after the incorporation of the Cu_4_I_4_ motif. However, the amount of generated hydrogen is very low (less than 0.5 µL of H_2_), indicating negligible hydrogen evolution. Moreover, the amount of generated hydrogen is much lower in the presence of nitrate. Such significantly suppressed hydrogen evolution suggests the preferential utilization of photogenerated electrons and reactive hydrogen species for nitrate reduction rather than hydrogen evolution.

**FIGURE 3:**
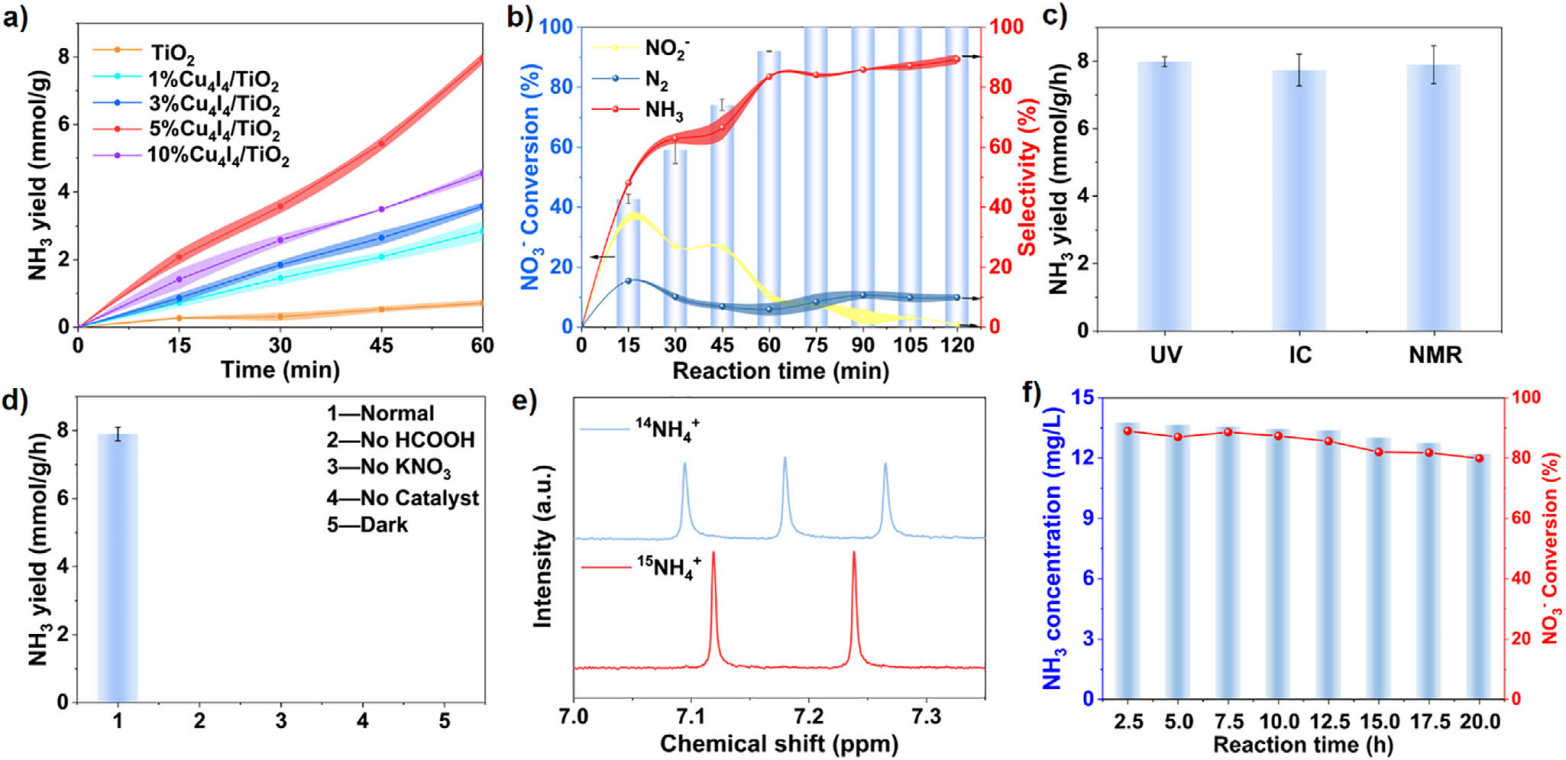
Photocatalytic nitrate reduction to ammonia performance. Time‐dependent plots of (a) ammonia yield and (b) ammonia selectivity and nitrate conversion over TiO_2_ and Cu_4_I_4_/TiO_2_ for photocatalytic nitrate reduction with HCOOH as sacrificial reagent. (c) Ammonia yield over Cu_4_I_4_/TiO_2_ using different detection methods. (d) Ammonia generation rate over Cu_4_I_4_/TiO_2_ under different experimental conditions. (e) ^1^H NMR spectra using ^15^NO_3_^−^ and ^14^NO_3_^−^ as the nitrogen source for photocatalytic nitrate reduction to ammonia over Cu_4_I_4_/TiO_2_. (f) Cycle tests of Cu_4_I_4_/TiO_2_ for photocatalytic nitrate reduction to ammonia.

To confirm the reliability of ammonia quantification, the product yields over 5%Cu_4_I_4_/TiO_2_ were cross‐validated using colorimetry, ion chromatography, and nuclear magnetic resonance (NMR) in Figures 3c and S26–S27. The control experiments under various conditions were carried out to rule out contamination from external nitrogen sources and demonstrate that ammonia generation is exclusively driven by photocatalytic nitrate reduction (Figures 3d and S23e). Furthermore, isotopic labeling experiments using ^15^NO_3_^−^ and ^14^NO_3_^−^ as nitrogen sources verify that the nitrogen in generated ammonia originates from nitrate, as evidenced by the ^1^H NMR signals of ^15^NH_4_^+^ doublet and ^14^NH_4_^+^ triplet peaks with coupling constants of 71.8 and 51.7 Hz, respectively (Figure 3e) [41]. The durability of the Cu_4_I_4_/TiO_2_ composite was evaluated through consecutive reaction cycles. As shown in Figure 3f, the ammonia yield remains nearly unchanged after 20 h, indicating its good reusability. In addition, XRD patterns and TEM images of the used catalysts exhibit no noticeable structural or morphological changes compared with the fresh sample (Figures S28 and S29). Moreover, the ICP‐OES measurements for the reaction solution were performed to evaluate the potential leaching during the reaction. As shown in Table S6, no detectable copper species are observed after the reaction, indicating negligible photocatalyst leaching under the reaction conditions. In addition, XPS spectra of the used catalyst exhibit similar profiles to those of the fresh catalyst (Figure S31), indicating the electronic structure of the catalyst is largely preserved after photocatalysis. Furthermore, the characteristic Lewis acid sites associated with copper species in the Cu_4_I_4_ motif (vide infra) remain intact in the pyridine adsorption infrared spectrum of the used catalyst (Figure S30), suggesting the retention of accessible copper active sites after the reaction. The good stability of the Cu_4_I_4_ photocatalyst is attributed to the structural robustness of the cubane Cu_4_I_4_ motif and the strong interaction of copper with iodide ions. These results highlight the robustness of the photocatalytic system and the advantage of operating under mild conditions, ensuring that the active sites remain structurally stable and confirming the reliability of the catalyst for photocatalytic nitrate reduction to ammonia.

To gain insights into the role of the Cu_4_I_4_ active center in enhancing photocatalytic performance, a series of photocatalysts comprising alternative copper species supported on TiO_2_ substrates, including Cu single atom (SA), Cu_2_I_2_ nanocluster, and CuI NP, were prepared. Particularly, an analogue of Cu_4_I_4_Py_4_ nanocluster, namely Cu_2_I_2_Py_4_ nanocluster, was synthesized and immobilized onto TiO_2_ for comparison. As reported previously, CuI tends to form a dimeric adduct of Cu_2_I_2_Py_4_ in the presence of excess pyridine [42], and the pyridine ligands can likewise be removed under mild calcination (Figure S32). Despite its compositional similarity, Cu_2_I_2_Py_4_ adopts a two‐dimensional rhombic Cu_2_I_2_ core rather than the three‐dimensional cubane geometry of Cu_4_I_4_ (Figure S33). Each Cu atom is coordinated with two iodide atoms and two pyridine ligands, leading to a planar coordination environment. Consequently, the Cu_2_I_2_/TiO_2_ composite features a flat Cu center, in contrast to the spatially distributed Cu center in Cu_4_I_4_/TiO_2_. Photocatalytic evaluation for nitrate reduction to ammonia in the presence of HCOOH as a sacrificial reagent reveals that all control catalysts, including Cu SA/TiO_2_, Cu_2_I_2_/TiO_2_, and CuI NP/TiO_2_, exhibit significantly lower ammonia yield (Figure S34). These results underscore the crucial structural advantages of the cubane‐type Cu_4_I_4_ architecture, whose low‐coordinated Cu sites, coupled with adjacent iodide atoms, play a decisive role in promoting efficient nitrate adsorption and hydrogenation to ammonia generation.

Encouraged by the superior photocatalytic performance, comprehensive characterizations were subsequently performed to elucidate the interfacial electronic structure and underlying mechanism over Cu_4_I_4_/TiO_2_. The incorporation of Cu_4_I_4_ nanocluster onto TiO_2_ significantly alters the surface chemistry of TiO_2_ by introducing highly exposed and low‐coordinated Cu active sites. Given that surface Lewis acidity plays a critical role in nitrate adsorption and activation [4], which is the key step in nitrate photoreduction to ammonia, temperature‐programmed desorption of ammonia (NH_3_‐TPD) was employed to assess the surface acidity. As shown in Figure 4a, the desorption peaks at 246°C and 360°C of Cu_4_I_4_/TiO_2_ correspond to ammonia adsorption on medium‐strong and strong acid sites, respectively. Compared with pristine TiO_2_, Cu_4_I_4_/TiO_2_ exhibits significantly stronger desorption signals across both regions, with the integrated peak area nearly doubling. This observation confirms that the incorporation of the Cu_4_I_4_ motif significantly enhances the surface acidity [43, 44]. Furthermore, as summarized in Table S7, the increment in ammonia desorption quantity correlates well with the loading amount of Cu_4_I_4_ nanocluster, confirming that the enhanced surface acidity primarily originates from the low‐coordinated Cu centers intrinsic to the Cu_4_I_4_ motif. To further identify the nature of acid sites, pyridine adsorption infrared spectroscopy (Py‐IR) was conducted (Figure 4b). The absorption bands at 1444 and 1604 cm^−1^ are characteristic of pyridine adsorbed on Lewis acid sites, while the band at 1575 cm^−1^ corresponds to Brønsted acid sites. The weak feature at 1494 cm^−1^ arises from overlapping contributions of both acid types. As summarized in Table S8, the ratio of Brønsted and Lewis acid sites (B/L) is very low for other Cu‐based photocatalysts, confirming the predominance of Lewis acidity [45]. Remarkably, Cu_4_I_4_/TiO_2_ displays a distinct absorption band at 1483 cm^−1^, characteristic of Lewis acid sites [45]. In contrast, this feature is absent in TiO_2_, Cu SA/TiO_2_, and CuI NP/TiO_2_ (Figures 4b and S35), suggesting the newly generated Lewis acid sites originate from the low‐coordinated Cu centers in the Cu_4_I_4_ nanocluster. This observation is consistent with the structural evolution of the Cu_4_I_4_ motif, in which the removal of pyridine ligands during the mild calcination exposes coordinatively unsaturated Cu sites that display a strong tendency to re‐coordinate with pyridine molecules, confirming their intrinsic Lewis acidity. These findings demonstrate that Cu_4_I_4_ nanocluster generates a large density of unsaturated Cu‐based Lewis acid sites.

**FIGURE 4:**
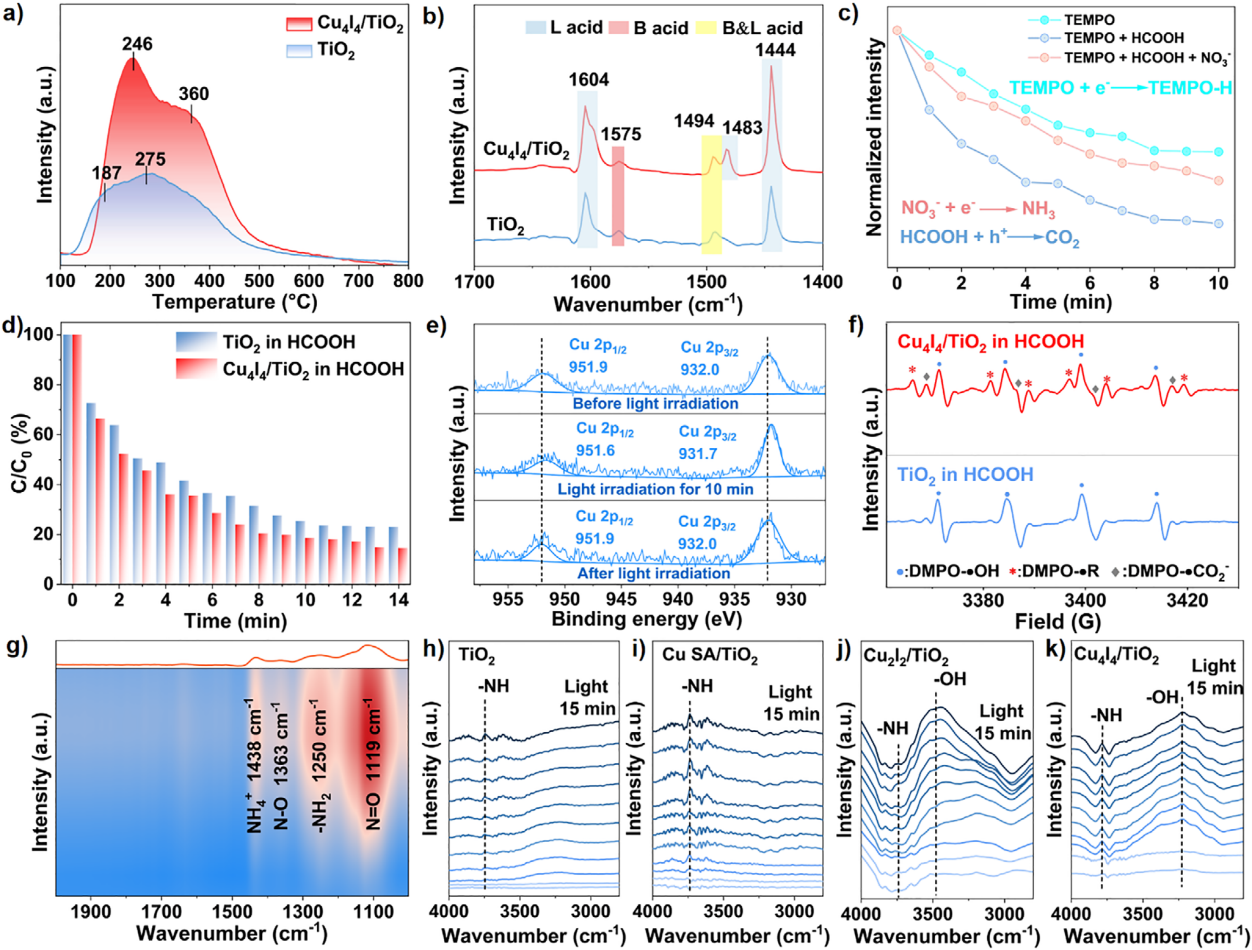
Mechanistic investigations of photocatalytic nitrate reduction reaction over Cu_4_I_4_/TiO_2_. (a) NH_3_‐TPD profiles and (b) Py‐IR spectra of TiO_2_ and Cu_4_I_4_/TiO_2_. (c) Evolution of the in situ EPR peak with normalized intensities of TEMPO over Cu_4_I_4_/TiO_2_ under varied reaction conditions. (d) In situ EPR spectra of TEMPO concentration consumption for HCOOH oxidative half‐reactions over TiO_2_ and Cu_4_I_4_/TiO_2_. (e) In situ Cu 2p XPS spectra of Cu_4_I_4_/TiO_2_ under varied irradiation conditions. (f) In situ EPR spectra of HCOOH oxidative half‐reactions over TiO_2_ and Cu_4_I_4_/TiO_2_ after 10 min of irradiation. (g) In situ ATR‐FTIR spectra of nitrate reduction over Cu_4_I_4_/TiO_2_ in the range of 1000–2000 cm^−1^. In situ ATR‐FTIR spectra for nitrate reduction over (h) TiO_2_, (i) Cu SA/TiO_2_, (j) Cu_2_I_2_/TiO_2_, and (k) Cu_4_I_4_/TiO_2_ in the range of 2800–4000 cm^−1^.

After identifying the atomic configuration of the active sites in Cu_4_I_4_/TiO_2_, (photo)electrochemical measurements were performed to corroborate the promotional effect of the Cu_4_I_4_ nanocluster in facilitating charge separation and transfer. As shown in Figure S36a, electrochemical impedance spectroscopy (EIS) for Cu_4_I_4_/TiO_2_ exhibits a smaller semicircular arc in the high‐frequency region than that of pristine TiO_2_, manifesting a reduced interfacial charge transfer resistance and more efficient electron transport [46]. In addition, the transient photocurrent response measurements reveal that Cu_4_I_4_/TiO_2_ generates the highest photocurrent density (Figure S36b), confirming that the incorporation of Cu_4_I_4_ nanocluster accelerates photoinduced charge separation. The enhancement is attributed to the improved photon‐harvesting efficiency and molecular‐level dispersion of Cu_4_I_4_ nanoclusters on TiO_2_, which maximizes the accessibility of active sites and interfacial charge transfer. Time‐resolved photoluminescence (TRPL) results show a shortened emission lifetime for Cu_4_I_4_/TiO_2_ compared with pristine TiO_2_, indicating more efficient separation of photogenerated charge carriers (Figure S36c) [47].

Given the decisive role of photogenerated carriers in determining the photocatalytic pathway, in situ electron paramagnetic resonance (EPR) measurements were performed to elucidate the interfacial redox dynamics during photocatalytic nitrate reduction to ammonia. First, in situ EPR experiments employing 2,2,6,6‐tetramethyl‐1‐piperidinyloxy (TEMPO) as an electron‐trapping agent were performed to record the behavior of photogenerated electrons [48]. As shown in Figure 4c, the addition of HCOOH accelerates the TEMPO consumption relative to pure water over Cu_4_I_4_/TiO_2_ as HCOOH scavenges photogenerated holes. Conversely, further introduction of nitrate into the system markedly suppresses TEMPO consumption, demonstrating that the photogenerated electrons are instead consumed in the nitrate reduction process. Given that the total electron population under illumination remains constant, the diminished rate of TEMPO consumption directly verifies that nitrate serves as the principal electron acceptor in the reduction reaction, further confirming that ammonia formation originates from photocatalytic electron‐driven processes. Similar trends are observed over pristine TiO_2_, where the synergistic oxidation of HCOOH accelerated the TEMPO consumption compared with water oxidation (Figure S37). However, Cu_4_I_4_/TiO_2_ exhibits an even higher TEMPO consumption rate in the presence of HCOOH than that of pristine TiO_2_ (Figure 4d), indicating that the incorporation of low‐coordinated Cu_4_I_4_ nanoclusters effectively promotes photoinduced electron generation and utilization. To further demonstrate the interfacial charge transfer mechanism in the Cu_4_I_4_/TiO_2_ photocatalyst, in situ XPS measurements under illumination were conducted. As displayed in Figure S38, compared with the XPS spectra acquired in the dark, the Ti 2p peaks of Cu_4_I_4_/TiO_2_ exhibit an obvious positive binding energy shift of 0.5 eV upon photoexcitation. Meanwhile, the Cu 2p peaks shift toward lower binding energy by 0.3 eV (Figure 4e). Considering the correlation between binding energy shift and electron density, the positive shift of Ti 2p peaks and the negative shift of Cu 2p peaks under illumination suggest a decrease in electron density at the Ti site and a corresponding electron accumulation at the Cu site. These observations confirm that photoinduced electrons transfer from the TiO_2_ support to the interfacial Cu_4_I_4_ active sites, facilitating the subsequent reduction of the surface‐adsorbed nitrate species. Notably, these shifts are fully reversible when the photocatalyst returns to dark conditions, indicating the structural stability and dynamic charge redistribution.

The generation of active radicals was further probed using 5,5‐dimethyl‐1‐pyrroline‐N‐oxide (DMPO) as a radical‐trapping agent. As shown in Figure 4f, when HCOOH serves as the oxidation substrate, TiO_2_ exhibits a distinct DMPO─•OH signal accompanied by a weak DMPO─•CO_2_^−^ signal, originating from the oxidation of water and HCOOH by photogenerated holes. These highly oxidative •OH radicals are known to impede selective nitrate reduction to ammonia by promoting potential reoxidation of intermediates [49]. In contrast to pristine TiO_2_, the DMPO─•OH signal is markedly attenuated over Cu_4_I_4_/TiO_2_ under the identical conditions, indicating a preferential oxidation of HCOOH over water, which effectively mitigates the generation of strongly oxidative radicals and prevents the undesired back‐oxidation process. Moreover, a distinct hyperfine coupling pattern, characteristic of organic radicals (DMPO─•R) that favors the transformation of nitrate to ammonia [50], is observed for Cu_4_I_4_/TiO_2_. These results provide direct evidence that the low‐coordinated Cu sites within the Cu_4_I_4_ motif facilitate the activation of HCOOH via hole‐driven oxidation to generate organic intermediates rather than •OH species. Therefore, HCOOH serves as a sacrificial reagent with a dual role in the photocatalytic nitrate reduction process. As a typical hole scavenger, HCOOH facilitates charge separation by rapidly consuming photogenerated holes and thereby increasing the availability of electrons for nitrate reduction. Moreover, the preferential oxidation of formic acid mitigates the generation of •OH radicals, preventing the undesired reoxidation of ammonia and the intermediates.

To gain mechanistic insights into the nitrate reduction pathway and clarify the role of the Cu_4_I_4_ motif, in situ attenuated total reflection‐Fourier transform infrared spectroscopy (ATR‐FTIR) was performed. According to Lewis acid‐base theory, Cu_4_I_4_ Lewis acid sites preferentially interact with electron‐rich NO_3_^−^, leading to the accumulation of NO_3_^−^ on the surface of Cu_4_I_4_/TiO_2_. When NO_3_^−^ is used as the sole reactant (Figure S39), distinct peaks at 1281 and 1430 cm^−1^, corresponding to NO_3_^−^ vibrations [51], confirm the adsorption of NO_3_^−^ on the catalyst surface. Under prolonged irradiation, gradual intensification of the bands at 1110 cm^−1^ (N═O stretching of NO_2_^−^) and 1350 cm^−1^ (N─O stretching of NO) implies the stepwise reduction sequence of NO_3_^−^ → NO_2_^−^ → NO on the Cu_4_I_4_/TiO_2_ catalyst [52]. However, characteristic peaks attributed to NH_3_ are scarcely detected due to the incomplete reaction under these conditions. In contrast, during HCOOH oxidation (Figure S40), the emergence and progressive growth of C─OH (1103 cm^−1^), ─OCO (1245 cm^−1^), ─C═O (1360 cm^−1^), and ─COOH (1430 cm^−1^) vibrations indicate efficient HCOOH activation and the accumulation of oxidation intermediates [53, 54, 55]. Remarkably, when NO_3_^−^ and HCOOH are introduced simultaneously (Figure 4g), both NO_3_^−^ adsorption and HCOOH oxidation features are intensified, accompanied by the rapid appearance of *NH_2_ (1250 cm^−1^), *NH_2_OH (1520 cm^−1^), NH_4_^+^ (1438 cm^−1^), and *N−O (1363 cm^−1^) absorption bands that are the characteristic of NH_3_ formation [56, 57]. These intermediates imply that Cu_4_I_4_/TiO_2_ speculatively follows the *NH_2_OH pathway (*NO → *NH_2_OH → *NH_2_ → *NH_3_). Moreover, these observations demonstrate that coupling NO_3_^−^ reduction with HCOOH oxidation substantially accelerates the selective conversion of NO_3_^−^ to NH_3_. Overall, Cu_4_I_4_/TiO_2_ and other catalysts display similar spectral features with comparable peak positions (Figures S41 and S42), but the absorption intensities are stronger for Cu_4_I_4_/TiO_2_. Specifically, although the absorption band at 1520 cm^−1^ belonging to *NH_2_OH is observed on both Cu_4_I_4_/TiO_2_ and TiO_2_, the *NH_2_‐related band at 1250 cm^−1^ is substantially stronger on Cu_4_I_4_/TiO_2_. This enhancement indicates that the incorporation of Cu_4_I_4_ nanocluster enhances the adsorption and activation of NO_3_^−^, thereby accelerating the transformation and deep hydrogenation of NH_3_ intermediates.

Apart from the adsorption and activation of nitrate during the photocatalytic ammonia synthesis, the hydrogenation process is equally critical, and sufficient *H provision is a prerequisite to sustain the selective ammonia formation [58]. To gain insights into this process, the interfacial water behavior is further examined using in situ ATR‐FTIR spectroscopy. As shown in Figure 4h–k, a broad adsorption band in the range of 3000–4000 cm^−1^, attributed to the O─H stretching vibration of adsorbed water molecules, is clearly observed for Cu_4_I_4_/TiO_2_ and Cu_2_I_2_/TiO_2_ [59]. In sharp contrast, this feature is barely detectable for TiO_2_ and Cu SA/TiO_2_, indicating that the presence of iodide species markedly enhances interfacial water adsorption and activation. Such enhancement can be rationalized by the formation of a moderate hydrogen bond between iodide anions and water molecules, which stabilizes surface‐bound water and promotes its activation. Moreover, the water bending vibration band observed at ∼1632 cm^−1^, which shifts to a lower wavenumber compared with pristine TiO_2_, confirms the presence of hydrogen‐bonded interfacial water (Figure S41) [60]. Such interactions not only strengthen water adsorption but also promote the *H generation. This is further validated via EPR spectroscopy using DMPO as the *H trapping agent. As shown in Figure 5a, Cu_4_I_4_/TiO_2_ exhibits stronger DMPO‐trapped *H signals than those of TiO_2_ and Cu SA/TiO_2_, confirming its superior H_2_O dissociation capability and abundant *H for the hydrogenation of nitrate on Cu_4_I_4_/TiO_2_. Considering the proximity of copper and iodide atoms within the Cu_4_I_4_ motif, the abundant reactive hydrogen species generated at the interface can be promptly utilized, thereby mitigating the intrinsically sluggish *H‐migration kinetics. Kinetic isotope effect (KIE) experiments using H_2_O and D_2_O as solvents were further performed to elucidate the *H generation and utilization behavior during nitrate photoreduction. As shown in Figure 5b, the ammonia concentration decreases for all samples when D_2_O is used as the solvent, indicating the involvement of protons from water dissociation. The KIE value is calculated based on the ratio of the reaction rate constant *k*_H_ in H_2_O to *k*_D_ in D_2_O. TiO_2_ and Cu SA/TiO_2_ exhibit KIE values of 1.92 and 1.61, respectively (Figure 5c). In contrast, Cu_4_I_4_/TiO_2_ delivers a KIE value of 1.20 under identical conditions. Such reduced isotope effect confirms that the generation and utilization of reactive hydrogen are favorable on Cu_4_I_4_/TiO_2_, enabled by the iodide active site and its proximity to the Cu site in the Cu_4_I_4_ motif. Therefore, the enhanced availability and utilization of *H ensure efficient stepwise hydrogenation of nitrate to ammonia. These results highlight the critical role of iodide within the Cu_4_I_4_ motif in facilitating the hydrogenation process by promoting the interfacial adsorption and enhancing its subsequent dissociation to generate reactive hydrogen species.

**FIGURE 5:**
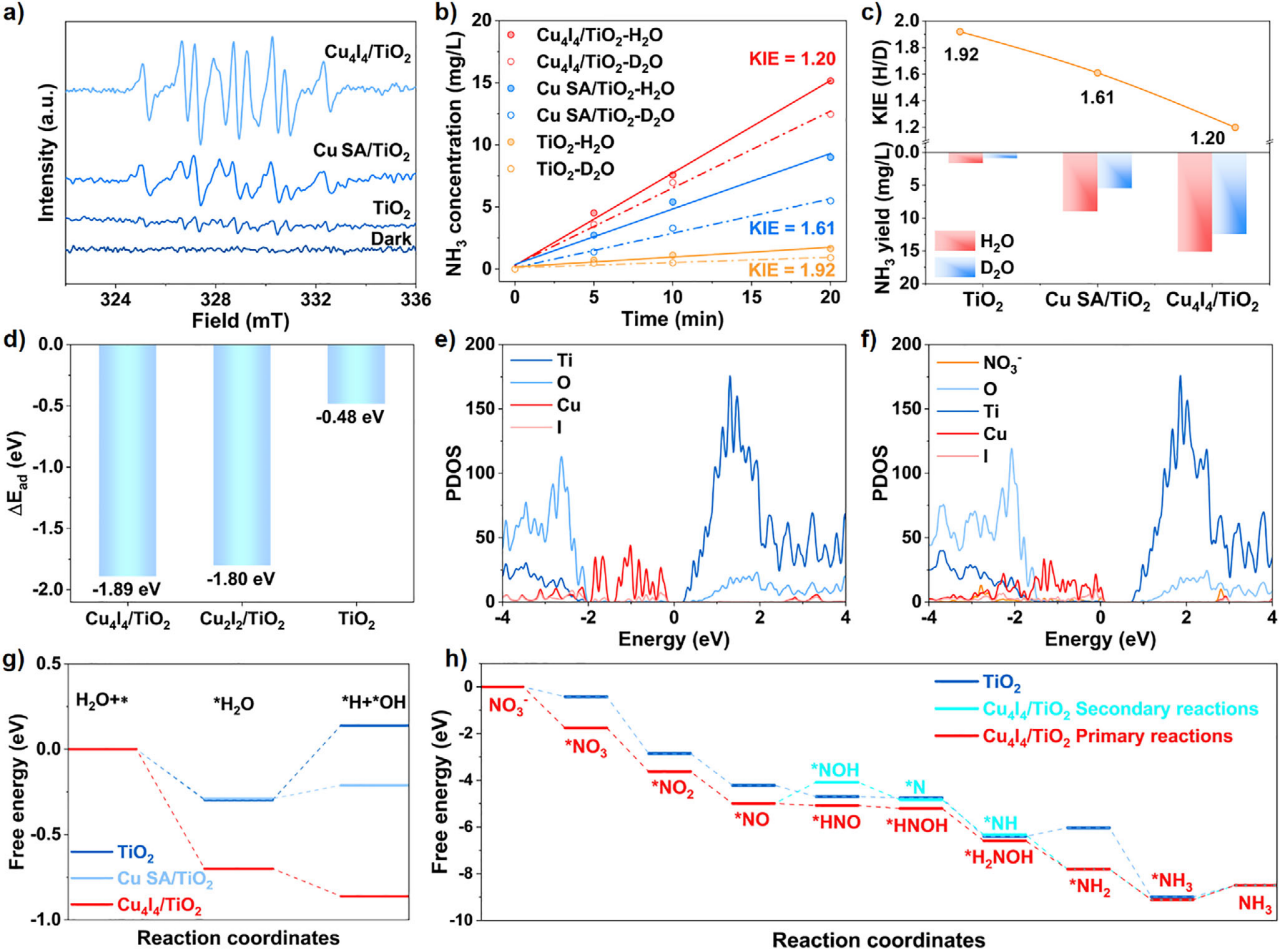
Mechanistic investigations and DFT calculations for the photocatalytic nitrate reduction mechanism. (a) EPR spectra of DMPO‐trapped *H over TiO_2_, Cu SA/TiO_2_, and Cu_4_I_4_/TiO_2_ in pure water. (b) Time‐dependent NH_3_ concentration and linear fitting line of TiO_2_, Cu SA/TiO_2_, and Cu_4_I_4_/TiO_2_ for photocatalytic nitrate reduction with H_2_O or D_2_O as solvent. (c) Related KIE and the NH_3_ yields of TiO_2_, Cu SA/TiO_2_, and Cu_4_I_4_/TiO_2_ for photocatalytic nitrate reduction with H_2_O or D_2_O as the solvent. Reaction conditions: 2 mg of catalyst, 5 mL of reaction solution (100 ppm nitrate, 2% formic acid), 300 W Xenon lamp. (d) Adsorption energies of nitrate on TiO_2_, Cu_2_I_2_/TiO_2_, and Cu_4_I_4_/TiO_2_. PDOS of Cu_4_I_4_/TiO_2_: (e) before and (f) after nitrate adsorption. (g) Gibbs free energy diagram of water adsorption and dissociation on TiO_2_, Cu SA/TiO_2_, and Cu_4_I_4_/TiO_2_. (h) Gibbs free energy diagram of the nitrate‐to‐ammonia on TiO_2_ and Cu_4_I_4_/TiO_2_.

Density functional theory (DFT) calculations were further performed to elucidate the role of the Cu_4_I_4_ motif in modulating the nitrate reduction mechanism. The optimized Cu_4_I_4_/TiO_2_ model shows that Cu atoms are coordinated to surface O atoms of TiO_2_, consistent with the experimental results. We first examine the adsorption behavior of nitrate on different catalyst surfaces (Figure S43). The calculated adsorption energies of nitrate on the TiO_2_, Cu_2_I_2_/TiO_2_, and Cu_4_I_4_/TiO_2_ are −0.48, −1.80, and −1.89 eV, respectively (Figure 5d), indicating substantially stronger nitrate binding on the Cu‐based surface. Compared with Cu_2_I_2_/TiO_2_, the enhanced adsorption on Cu_4_I_4_/TiO_2_ could be ascribed to reduced steric hindrance associated with the cubane structure of Cu_4_I_4_/TiO_2_. Charge density difference results reveal that nitrate acts as an electron acceptor on all three catalysts (Figure S44). Bader charge analyses show charge transfers of 0.22, 0.51, and 0.61 e^−^ from TiO_2_, Cu_2_I_2_/TiO_2_, and Cu_4_I_4_/TiO_2_ to nitrate, respectively. Moreover, projected density of state (PDOS) analyses further provide insights into the electronic interactions (Figures 5e and S45). Upon Cu_4_I_4_ incorporation, new electronic states emerge near the Fermi level, and significant hybridization of Cu orbitals with I and O orbitals is observed, suggesting the stable anchoring of the Cu_4_I_4_ nanocluster on TiO_2_. After nitrate adsorption, additional states are introduced at the Fermi level for both TiO_2_ and Cu_4_I_4_/TiO_2_ (Figure 5f). Notably, the strong hybridization between nitrate orbitals and Cu *d*‐orbitals in Cu_4_I_4_/TiO_2_ implies effective electronic coupling. The increased electron donation and strong electronic coupling facilitate nitrate activation and subsequent reduction steps. Besides, water adsorption and dissociation were evaluated on TiO_2_, Cu SA/TiO_2_, and Cu_4_I_4_/TiO_2_ (Figures 5g and S46). Cu_4_I_4_/TiO_2_ exhibits favorable energetics for both water adsorption and dissociation, suggesting the efficient supply of reactive hydrogen during nitrate reduction.

Furthermore, the energetics associated with the elementary steps from nitrate to ammonia were systematically evaluated on TiO_2_ and Cu_4_I_4_/TiO_2_. As shown in Figures 5h and S47–S49, Gibbs free energy diagrams reveal that the initial deoxygenation steps from *NO_3_ to *NO on Cu_4_I_4_/TiO_2_ have a much lower energy barrier than that of pristine TiO_2_, indicating facilitated nitrate activation. Subsequently, there are two possible hydrogenated pathways, leading to either *HNO or *NOH. The calculated free energy change for *HNO (Δ*G* = −0.09 eV) is substantially lower than that for *NOH formation (Δ*G* = 0.91 eV). Therefore, the plausible pathway of nitrate reduction to ammonia is proposed as *NO_3_ → *NO_2_ → *NO →*HNO → *HNOH → *H_2_NOH → *NH_2_ → *NH_3_, consistent with the ATR‐FTIR results. Importantly, the preferential generation of *HNO suppresses the formation of *N intermediates, thereby avoiding *N–*N coupling that will favor dinitrogen formation. Notably, the subsequent hydrogenation steps from *HNO to *NH_3_ are thermodynamically downhill on Cu_4_I_4_/TiO_2_. In contrast, on pristine TiO_2_, the transformation from *H_2_NOH to *NH_2_ is energetically unfavorable (Δ*G* = 0.35 eV), representing a key bottleneck for the deep hydrogenation. This is in good agreement with the observation in ATR‐FTIR spectra (Figure S41), where the generated *NH_2_OH intermediate is hardly reduced to *NH_2_ for TiO_2_. Although the free‐energy change for NH_3_ desorption slightly increases after Cu_4_I_4_ modification (0.61 vs. 0.54 eV for pristine TiO_2_), the difference is relatively small. More importantly, Cu_4_I_4_ effectively eliminates the thermodynamic penalty associated with the hydrogenation process on pristine TiO_2_, rendering all hydrogenation steps thermodynamically downhill. Collectively, these results demonstrate that the incorporation of Cu_4_I_4_ nanocluster onto TiO_2_ facilitates the deep hydrogenation of nitrate‐to‐ammonia. This enhancement plausibly originates from the synergistic functions of the Cu_4_I_4_ motif, which simultaneously strengthens nitrate adsorption and activation while promoting interfacial hydrogen generation to drive multistep hydrogenation efficiently.

## 3 Conclusion

In summary, we demonstrate that a Cu_4_I_4_ nanocluster, when anchored onto TiO_2_, acts as a robust and highly active center for the photocatalytic nitrate reduction to ammonia. The photocatalyst is constructed based on an atomically precise Cu_4_I_4_Py_4_ nanocluster under a mild thermal treatment to remove labile pyridine ligands. The ligand‐stripped Cu_4_I_4_ motif is anchored onto TiO_2_ via Cu–O interactions, giving rise to a multifunctional catalytic interface with highly exposed, low‐coordinated Cu Lewis acid sites in proximity to iodide ligands. This unique configuration offers a plausible basis for optimizing the key processes governing the nitrate reduction to ammonia. Specifically, the low‐coordinated Cu centers with strong Lewis acidity preferentially capture and activate nitrate. Concurrently, the adjacent iodide ligands are suggested to promote interfacial water activation and *H provision, effectively alleviating the intrinsically sluggish *H migration kinetics. This cooperative Cu–I functionality is expected to ensure abundant reactive hydrogen species, which promptly participate in the multistep hydrogenation toward NH_3_. Consequently, the Cu_4_I_4_/TiO_2_ photocatalyst delivers remarkable performance in a sacrificial‐reagent‐assisted photocatalytic nitrate reduction system, achieving a high ammonia yield rate of 26.8 mmol·g_cat_^−1^·h^−1^ and a conversion of ∼100%, ranking among the state‐of‐the‐art photocatalytic systems for nitrate reduction to ammonia. Such efficient nitrate reduction half‐reaction provides an essential foundation for coupling selective oxidation of organic substrates into value‐added chemicals or for achieving overall photocatalytic nitrate conversion without sacrificial reagent via Z‐scheme systems, where two photocatalysts are responsible for the reduction and oxidation half‐reactions, respectively. Combined experimental and computational investigations support the decisive role of the cubane Cu_4_I_4_ configuration in modulating interfacial electronic structure, adsorption energetics, and reaction pathways. These findings not only deepen the mechanistic understanding of interfacial chemistry in photocatalysis but also open a promising avenue for atomic‐level engineering of atomically precise nanoclusters as a versatile strategy for the rational design of multifunctional catalytic interfaces and systems.

## Author Contributions

**Wanting Zhang**: investigation, writing – original draft, methodology, formal analysis. **Yuehua Li**: investigation, visualization, writing – review and editing. **Junjie Jin**: investigation, writing – review and editing, methodology. **Chunwei Dong**: investigation, writing – review and editing, formal analysis. **Xi Cao**: investigation, visualization, writing – review and editing. **Xingchi Li**: investigation, writing – review and editing, formal analysis, validation. **Xuanjun Wu**: writing – review and editing, investigation, methodology. **Jiaqi Wang**: investigation, methodology, writing – review and editing. **Atta Ullah**: investigation, writing – review and editing, formal analysis. **Gaspar Ferreyra Vaggione**: formal analysis, writing – review and editing, investigation. **Rodrigo A. Iglesias**: supervision, writing – review and editing, funding acquisition. **Yun Chen**: investigation, writing – review and editing, data curation. **Emiliano Cortés**: resources, supervision, project administration, writing – review and editing, conceptualization, funding acquisition. **Dengsong Zhang**: conceptualization, funding acquisition, writing – original draft, project administration, data curation, supervision, resources.

## Conflicts of Interest

The authors declare no conflicts of interest.

## Supporting information




**Supporting File**: The detailed experimental section, additional figures, and tables are listed in the Supporting Information. The authors have cited additional references within the Supporting Information [61, 62, 63, 64, 65, 66, 67, 68, 69, 70, 71, 72].

## Data Availability

The data that support the findings of this study are available from the corresponding author upon reasonable request.
