# Supplementary material for: Engineering Atomically Precise Cu4I4 Nanoclusters With Integrated Adsorption and Hydrogenation Functions for Efficient Photocatalytic Nitrate‐to‐Ammonia Conversion
Source: Angew Chem Int Ed Engl. 2026 Aug 29;65(43):e5529008. doi: 10.1002/anie.5529008 (PMC13676402; doi:10.1002/anie.5529008)
Supplement: Supplementary file 1 [file ANIE-65-e5529008-s001.docx]

**Supporting Information**

Engineering Atomically Precise Cu_4_I_4_ Nanoclusters with Integrated Adsorption and Hydrogenation Functions for Efficient Photocatalytic Nitrate to Ammonia Conversion

Wanting Zhang,^[a]+^ Yuehua Li,^[a]+^ Junjie Jin,^[c]+^ Chunwei Dong,*^[a]^ Xi Cao,^[a]^ Xingchi Li,^[a]^ Xuanjun Wu,^[a]^ Jiaqi Wang,^[a]^ Atta Ullah,^[a]^ Gaspar Ferreyra Vaggione,^[b,e]^ Rodrigo A. Iglesias,^[e]^ Yun Chen,^[d]^ Emiliano Cortés,*^[b]^ Dengsong Zhang*^[a]^

[a]	International Joint Laboratory of Catalytic Chemistry, Innovation Institute of Carbon Neutrality, Department of Chemistry, College of Sciences, Shanghai University, Shanghai 200444, China

[b]	Nanoinstitute Munich, Faculty of Physics, Ludwig-Maximilians-Universität (LMU), Munich 80539, Germany

[c]	School of Physics and Electronic Information Engineering, Guangxi Minzu Normal University, Chongzuo, Guangxi 532200, China

[d]	Department of Physical Chemistry, School of Chemistry, Tel Aviv University, Tel Aviv 6997801, Israel

[e]	INFIQC CONICET, Departamento de Fisicoquímica, Facultad de Ciencias Químicas, Universidad Nacional de Córdoba (UNC), X5000HUA Córdoba, Argentina.

^+^These authors contribute equally to this work.

*Corresponding authors: chunwei_dong@shu.edu.cn (C. Dong), Emiliano.Cortes@lmu.de (E. Cortés), dszhang@shu.edu.cn (D. Zhang).

**Experimental Procedures**

**Materials**

All chemicals were purchased from commercial suppliers and used as received without further purification (**Table S1**).

**Table S1**. Source and purity of chemicals used in this work.

| Chemicals | Purity | Source |
| --- | --- | --- |
| CuI | 99.5% | Aladdin |
| Acetonitrile | 99.9% | Macklin |
| Acetone | AR | Sinopharm Chemical Reagent |
| Ethanol | AR | Sinopharm Chemical Reagent |
| Potassium nitrate | 99% | Aladdin |
| TiO_2_ | 99.9% | Degussa |
| Formic acid | 88% | Aladdin |
| 2,9-dimethyl-l,10-phenanthroline | 98% | Aladdin |
| 2,2,6,6­tetramethyl-1-piperidinyloxy | 99% | Aladdin |
| Potassium nitrate ^15^N | 99% | Aladdin |
| Ammonium chloride | AR | Sinopharm Chemical Reagent |
| Maleic acid | AR | Shanghai Yuanye |

**Catalysts Preparation**

**Synthesis of Cu_4_I_4_Py_4_.** For the synthesis of Cu_4_I_4_Py_4_, CuI (1 mmol) was dissolved in 10 mL of acetonitrile. Then, 100 μL of pyridine was added to the mixture and thoroughly homogenized. Finally, deionized (DI) water was introduced to induce precipitation and crystal formation. It was washed repeatedly with ethanol (EtOH) and DI water followed by centrifugation, then dried at 25 °C for 2 hours, and labeled as the Cu_4_I_4_Py_4_.

**Synthesis of Cu_4_I_4_/TiO_2_.** A certain amount of Cu_4_I_4_Py_4_ was ultrasonically dispersed in 20 mL of acetone to ensure complete dissolution. TiO_2_ (Commercial P25) was added into the solution and stirred at room temperature until complete evaporation of acetone. The mixture was transferred into a quartz boat, which was placed in a tube furnace at a heating rate of 5 °C/min and maintained at 140 °C for 1 hour under an argon (Ar) atmosphere. Above sample was labeled as the 5%Cu_4_I_4_/TiO_2_. The nominal weight percentages of Cu_4_I_4_Py_4_ and TiO_2_ were 1%, 3%, 5%, and 10%, and the samples were labeled as 1%Cu_4_I_4_/TiO_2_, 3%Cu_4_I_4_/TiO_2_, 5%Cu_4_I_4_/TiO_2_, and 10%Cu_4_I_4_/TiO_2_, respectively.

**Evaluation of Photocatalytic Activity**

In a typical photocatalytic test, 5 mg of catalyst powder was dispersed in 50 mL of reaction solution containing 100 mg/L potassium nitrate (K) and 2%HCOOH. Then, the mixture was transferred into a double-walled quartz reactor and purged with Ar for 30 min. The light source is a 300 W Xe lamp (MC-GF250, Merry Change, China) with full spectrum. The light intensity was measured to be 0.83 W/cm^2^, using a photoradiometer (PL-MW2000, Beijing Perfectlight Co., Ltd.). The temperature was controlled at room temperature by using a water circulation system. The analysis of NO_3_^−^, nitrite (NO_2_^−^), and ammonia (NH_3_) was performed by ion chromatography (IC, Compact IC-930 equipped with Metrosep C 6-250/4.0 for cations and Analysis Lab equipped with Metrosep A Supp 19-250/4.0 for anions). The NH_3_ yield was quantified via spectrophotometric measurement based on the indophenol blue method.

Specifically, for the indophenol blue method, a defined volume of the post-irradiation reaction solution was first diluted to fall within the detection range. Subsequently, 2 mL of the diluted reaction solution was thoroughly mixed with 2 mL of Reagent A, 1 mL of Reagent B, and 200 μL of Reagent C. Reagent A consisted of a 1 M sodium hydroxide (NaOH) solution containing 5 wt.% salicylic acid and 5 wt.% trisodium citrate dihydrate, respectively. Reagent B was a 0.05 M sodium hypochlorite solution, while Reagent C was a 1 wt.% sodium nitroprusside solution. The resultant mixture was incubated at ambient temperature for 1 h, followed by absorbance measurement at 655 nm using a Cary5000 UV-visible (UV-vis) spectrophotometer (Agilent Technologies, USA). A calibration curve was established using a series of ammonium chloride (NH_4_Cl) standard solutions for accurate NH_3_ quantification.

For isotope labeling experiments, ^15^NO_3_^−^ was employed as the nitrogen source. The ^1^H nuclear magnetic resonance (^1^H NMR) spectra were recorded on a Bruker 600 MHz spectrometer, with 20% deuterated dimethyl sulfoxide (DMSO-d6) serving as the solvent.

The cycling tests were performed to evaluate the catalytic stability, which involved 5 h of continuous light irradiation. During the tests, NO_3_^−^ was supplemented every 1 h to ensure a continuous supply of the nitrogen source. The selectivity of NH_3_ in the photocatalytic nitrate reduction process was calculated using the following equation:

$${Sel}_{NH_{3}-N}\left( \% \right)=\frac{\left[ NH_{3}-N \right]\times8}{\left[ NO_{2}^{-}-N \right]\times2+\left[ N_{2}-N \right]\times5+\left[ NH_{3}-N \right]\times8}\times100\%$$

Determination of apparent quantum yield (AQY):

Under monochromatic light (350 nm) irradiation, the value of light intensity was measured to 89.17 mW•cm^-2^. NH_3_ yield was measured after 10 min of irradiation, and AQY was calculated according to the following formula:

$$\begin{matrix} \mathrm{AQY} \left( \% \right)=\frac{Number of reacted electrons}{Number of incident photons}\times100\% & \\ =\frac{Number of evolved NH_{3} molecules\times8}{I\times S\times\left( \frac{\lambda t}{\mathrm{hc}} \right)}\times100\% & \end{matrix}$$

where *I* is the light intensity, $S$ is the light incident area (12.56 cm^2^), $\lambda$ is the wavelength, *t* is the irradiation time (10 min), ℎ is the Planck constant, and$c$ is the speed of light.

**Catalyst Characterization**

X-ray diffraction (XRD) measurements were conducted using a Bruker D8-Advance diffractometer equipped with a Cu Kα monochromator to characterize the crystal structures of the catalysts. High-resolution transmission electron microscopy (HR-TEM, JEM-F200) and aberration-corrected high-angle annular dark-field scanning transmission electron microscopy (AC-HAADF-STEM, JEM-ARM 300F Grand ARM) were applied to examine the morphological features of the catalysts. X-ray photoelectron spectroscopy (XPS) analysis was carried out using an AXIS Kratos supra spectrometer with Mg-Kα as the excitation source to investigate the sample compositions, and the obtained spectra were calibrated against the C 1s peak at 284.8 eV. X-ray absorption near-edge structure (XANES) and extended X-ray absorption fine structure (EXAFS) spectra were collected at the SSRF BL13SSW beamline at the Shanghai Synchrotron Radiation Facility. The EXAFS data were fitted and analyzed using the Athena and Artemis modules within the Demeter data analysis package, which employs the FEFF6 program for EXAFS fitting.

**The NH_3_ temperature programmed desorption (NH_3_-TPD) measurement**

The NH_3_-TPD tests were performed on Micromeritics Auto Chem HP 2920 instrument coupled with a mass spectrometry (OMNISTAR) to elucidate the acidity of the catalysts. Briefly, 80 mg of catalyst was loaded into a glass tube and pre-treated by Ar (30 mL/min) flow at 100 ºC for 30 min, followed by cooling to 30 ºC. The adsorption of NH_3_ was then conducted at 30 ºC. After purging with Ar for 30 min to remove the physical adsorption of NH_3_, the sample underwent heating at a rate of 10 ºC/min, increasing the temperature from 30 ºC to 800 ºC. The mass spectrometer (MS) signals corresponding to m/z = 17 were monitored and recorded using the OMNISTAR MS detector.

**Pyridine adsorption infrared (Py-IR) spectroscopy measurement**

The Py-IR measurements were carried out using a Nicolet 6700 spectrometer. To eliminate physical adsorption and surface contaminants, the samples underwent pretreatment at 100 °C for 0.5 h under vacuum. Subsequently, background spectra were collected at various target temperatures, which were utilized for subtraction from the sample spectra afterward. Afterward, the catalysts were exposed to pyridine vapor at ambient temperature for 10 min to facilitate sufficient adsorption. Finally, the temperature-programmed desorption process was performed, and the spectral data were collected at the corresponding temperatures.

**In situ electron paramagnetic resonance (EPR) measurement**

In situ EPR measurement was conducted on the EPR200-PLUS model equipment of China National Instrument Quantum Corporation. 2,9-dimethyl-l,10-phenanthroline (DMPO) was the trapping agent to trap radicals, forming longer-lived nitroxide spin adducts. 2,2,6,6­tetramethyl-1-piperidinyloxy (TEMPO) was utilized as the e^−^ trapping reagent. In a typical process, 1 mg catalyst and 400 µL trapping reagent solution (DMPO or 0.1 mol•L^−1^ of TEMPO) were added to 5 mL of the reaction solution. Then, a 300 W Xe lamp was applied as the light source. The spectra were generated using the following spectrometer operating conditions: test temperature, 25 °C; modulation frequency, 100 kHz; microwave power, 1 mW; microwave frequency, 9.52 GHz; scanning center, 3398 G; scanning width, 100 G; modulation amplitude, 1 G.

**In situ attenuated total reflection infrared absorption spectroscopy (ATR-IRAS) measurement**

In situ ATR-FTIR Tests were performed using a Bruker INVENIO R spectrometer equipped with a liquid nitrogen-cooled mercury cadmium telluride A (MCT-A) detector. The catalyst ink was a mixture of 10 mg of catalyst, 30 µL of Nafion, 360 µL of ethanol and 180 µL of DI water. The catalyst ink was evenly coated on the surface of face-angled silicon (Si) crystal. All spectra were expressed in terms of absorbance, defined as [-log(R/R_0_)]. The spectral resolution was set to 4 cm^−1^, with a scanning duration of 1 min per spectrum. Ar was pumped into the reaction system for 20 minutes to maintain the inert atmosphere. The signals were continuously detected during the adsorption process in dark and the reaction process with the light on.

**DFT calculations**

The first-principles calculations are performed by density functional theory (Vienna Ab-initio Simulation Package),^[61]^ and the generalized gradient approximation of Perdew-Burke-Ernzerhof is applied to the exchange-correlation functional. The van der Waals interaction is considered by means of the DFT-D3 method.^[62]^ The plane wave cutoff energy is set to 450 eV, and the total energy and atomic forces are converged to 10^−5^ eV and 0.02 eV/Å, respectively. A 3×3×1 k-mesh is utilized for Brillouin-zone integration. A vacuum region with a thickness larger than 15 Å is added in the z direction to avoid unphysical interaction. Charge transfer analysis is performed by the Bader method.^[63]^ The (101) plane of TiO_2_ is selected in this work. Free energy is calculated by G = E_total_ + E_ZPE_ – TS, where E_total_ is the total energy of substrate with adsorbed molecules, E_ZPE_ and TS are the zero-point energy correction, and the entropy change at room temperature. The free energy difference (ΔG) between the initial state (IS) and final state (FS) is calculated as: ΔG = G_FS_ – G_IS_.

The theoretical structures of Cu_4_I_4_/TiO_2_ and Cu_2_I_2_/TiO_2_ were constructed based on the crystal structures of the nanoclusters (Cu_4_I_4_Py_4_ and Cu_2_I_2_Py_4_) and then performed fully geometry optimization. The Cu_4_I_4_ and Cu_2_I_2_ motifs derived from Cu_4_I_4_Py_4_ and Cu_2_I_2_Py_4_ were anchored on the thermodynamically stable anatase TiO_2_ (101) supercell separately with a vacuum layer larger than 15 Å. During DFT geometry optimization, the nanocluster and surface atoms were fully relaxed, whereas the bottom atoms of TiO_2_ were constrained. The optimal configurations with the lowest formation energy were adopted to explore the photocatalytic nitrate reduction mechanism. For the photocatalytic nitrate reduction process, the full reaction pathway and Gibbs free energy profiles were systematically calculated for the Cu_4_I_4_/TiO_2_ system, which was mainly used to compare and reveal the intrinsic reaction difference.

**Figure S1.** The crystal structure of a) Cu_4_I_4_Py_4_ nanocluster and b) Cu_4_I_4_ cubane core.

**Figure S2.** The coordination environment of copper atoms in Cu_4_I_4_Py_4_ nanocluster. Each copper atom adopts a four-coordinated tetrahedral configuration.

**Figure S3.** XRD pattern of Cu_4_I_4_Py_4_.

**Figure S4.** Raman spectrum of Cu_4_I_4_Py_4_.

**Figure S5.** a) Emission spectra at varied excitation wavelengths and b) excitation and emission spectra of Cu_4_I_4_Py_4_.

**Figure S6.** XRD patterns of CuI and TiO_2_.

**Figure S7.** a) TEM and b) HR-TEM images for the Cu_4_I_4_/TiO_2_.

**Figure S8.** a) UV-vis DRS spectra for the TiO_2_ and Cu_4_I_4_/TiO_2_. b) The converted Kubelka-Munk function versus the optical energy for the TiO_2_.

**Table S2** Cu contents determined by ICP-OES for Cu_4_I_4_/TiO_2_ at different wt.% loadings.

| Sample | Cu (wt.%) |
| --- | --- |
| 1% Cu_4_I_4_/TiO_2_ | 0.2325 |
| 3% Cu_4_I_4_/TiO_2_ | 0.7214 |
| 5% Cu_4_I_4_/TiO_2_ | 0.9559 |
| 10% Cu_4_I_4_/TiO_2_ | 1.8943 |

**Figure S9.** XPS survey spectra of TiO_2_ and Cu_4_I_4_/TiO_2_.

**Figure S10.** Cu 2p and I 3d XPS spectra of CuI.

**Figure S11.** Fitting curve of Cu K-edge extended X-ray absorption fine structure spectrum of Cu_4_I_4_/TiO_2_ in a) R space and b) k space.

**Table S3** EXAFS fitting results of the Cu_4_I_4_/TiO_2_.

| Shell | CN | R(Å) | σ^2^ (10^-3^) | E_0_(eV) | R-factor | S_0_ |
| --- | --- | --- | --- | --- | --- | --- |
| Cu**–**O | 0.7 (0.4) | 2.03 (0.04) | 8.1 (0.9) | 6.5 (0.8) | 0.02 | 0.75 |
| Cu**–**Cu | 2.6 (0.3) | 2.58 (0.03) | 9.2 (0.8) | 1.1 (0.6) |  |  |
| Cu**–**I | 2.5 (0.5) | 2.63 (0.04) | 9.2 (0.8) | 1.1 (0.6) |  |  |

**Figure S12.** Standard curve for the detection of ammonia by colorimetry.

**Figure S13.** a) IC detection spectra of standard nitrate solutions. (b) Standard curve for the detection of nitrate by IC detection.

**Figure S14.** a) IC detection spectra of standard nitrite solutions. (b) Standard curve for the detection of nitrite by IC detection.

**Figure S15.** Selectivity and conversion tests for TiO_2_. (Reaction conditions: 5 mg catalyst, 2% FA, Time = 1 h, 300 W Xenon lamp).

**Figure S16.** a) The gas chromatograms of N_2_ with different volumes. b) The standard concentration curve of N_2_ obtained by gas chromatography.

**Figure S17.** a) The amount of produced N_2_ and b) the amount of nitrogen element in the photocatalytic nitrate reduction reactions over TiO_2_ and Cu_4_I_4_/TiO_2_.

**Figure S18.** Mass spectrum of the gaseous products after photocatalytic nitrate reduction reaction.

**Figure S19.** a) UV-vis absorption spectra of standard hydrazine solution by colorimetry. b) Standard concentration curve of hydroxylamine obtained by colorimetry. c) UV-vis absorption spectra for the photocatalytic reaction solutions of Cu_4_I_4_/TiO_2_ and TiO_2_ by colorimetry.

**Figure S20.** a) UV-vis absorption spectra of standard hydrazine solution by colorimetry. b) Standard concentration curve of hydroxylamine obtained by colorimetry. c) UV-vis absorption spectra for the photocatalytic reaction solutions of Cu_4_I_4_/TiO_2_ and TiO_2_ by colorimetry.

**Figure S21.** XRD patterns of Cu_4_I_4_/TiO_2_ with varied loading amounts of Cu_4_I_4_Py_4_.

**Figure S22.** XRD patterns of TiO_2_, CuI and 5%Cu_4_I_4_/TiO_2_ with different calcination temperatures. b) ammonia production yields for 5%Cu_4_I_4_/TiO_2_ with different calcination temperatures. (Reaction conditions: 5 mg catalyst, 2% FA, Time = 1 h, 300 W Xenon lamp).

**Figure S23.** a) Ammonia yield of photocatalytic nitrate reduction reaction for Cu_4_I_4_/TiO_2_ under different HCOOH concentrations (Reaction conditions: 5 mg catalyst, 2% FA, Time = 1 h, 300 W Xenon lamp). b) Ammonia yield of photocatalytic nitrate reduction reaction for Cu_4_I_4_/TiO_2_ under different light intensities (Reaction conditions: 5 mg catalyst, 2% FA, Time = 1 h, 300 W Xenon lamp). c) Ammonia production rate of Cu_4_I_4_/TiO_2_ at different nitrate concentrations (Reaction conditions: 2 mg catalyst, 2% FA, Time = 0.5 h, 300 W Xenon lamp). d) Ammonia yield of photocatalytic nitrate reduction reaction for Cu_4_I_4_/TiO_2_ under different pH (Reaction conditions: 2 mg catalyst, 2% FA, Time = 1 h, 300 W Xenon lamp). e) Ammonia generation rate over Cu_4_I_4_/TiO_2_ under different experimental conditions.

**Table S4.** The apparent quantum yield of photocatalytic ammonia production over Cu_4_I_4_/TiO_2_ at different monochromatic wavelengths.

| Wavelength, λ (nm) | light intensity, *I* (mW•cm^-2^) | NH_3_ yield (μmol) | AQY (%) |
| --- | --- | --- | --- |
| 350 | 89.17 | 4.77 | 1.95 |

**Table S5** Comparison of the results of photocatalytic nitrate-to-ammonia conversion with different photocatalysts.

| Photocatalyst | Illustration | Sacrificial agent | Optimal NH_4_^+^  synthesis rate, mmol•g_cat_^−1^•h^−1^ | NO_3_^−^ conversion ratio, % | Reference |
| --- | --- | --- | --- | --- | --- |
| Cu_4_I_4_/TiO_2_ | 300 W Xenon lamp | FA | 26.8 | 100 | This work |
| AgCu-CN | 300 W Xenon lamp | Ethylene glycol (EG) | 0.63 | Not provided | ^[4]^ |
| Cu-NM | 300 W Xenon lamp | EG | 1.93 | Not provided | ^[64]^ |
| CuO_x_@TNS | 300 W Xenon lamp | EG | 16.04 | 100 | ^[49]^ |
| BaO_NCs_-TNS | 300 W Xenon lamp | EG | 15.80 | 4.5 | ^[65]^ |
| Au@UiO-66 | 300 W Xenon lamp | - | 0.81 | Not provided | ^[66]^ |
| K^+^@g-C_3_N_4_ | 300 W Xenon lamp | EG | 3.42 | Not provided | ^[67]^ |
| JRC-TIO-6 | 300 W Xenon lamp | FA | 0.02 | 79 | ^[68]^ |
| Ag_2_O/P25 | High-pressure Hg lamp | FA | 0.45 | 83.4 | ^[69]^ |
| InP QDs | 20 W LED | - | 0.215 | 68 | ^[70]^ |
| CuPd/TiO_2_ | 300 W Xenon lamp | Methanol | 0.515 | 94.7 | ^[71]^ |
| Cu_2_O-TNS | 300 W Xenon lamp | Formaldehyde | 1.983 | 94.2 | ^[20]^ |
| GSH-QDs | 440 nm LED | Ascorbic acid and sodium ascorbate | 2.421 | Not provided | ^[72]^ |

**Figure S24.** a) The gas chromatograms of H_2_ with different volumes. b) The standard concentration curve of H_2_ obtained by gas chromatography.

**Figure S25.** a) The gas chromatograms and b) peak area of H_2_ over TiO_2_ and Cu_4_I_4_/TiO_2_ under different reaction conditions. Reaction conditions: 5 mg of catalyst, 15 mL of reaction solution (100 ppm of nitrate if necessary, 1.33% formic acid), 300 W Xenon lamp for 20 min irradiation.

**Figure S26.** a) IC detection spectra of standard ammonia solutions. b) Standard curve for the detection of ammonia by IC detection.

**Figure S27.** a) ^1^H NMR spectra of standard NH_4_^+^ solutions. b) Standard curve for ^14^NH_4_^+^ detection using ^1^H NMR, where ^14^NH_4_^+^ peak area integrals were normalized to that of maleic acid (C_4_H_4_O_4_).

**Figure S28.** XRD spectra of fresh Cu_4_I_4_/TiO_2_ and used Cu_4_I_4_/TiO_2_.

**Figure S29.** TEM images of a) fresh Cu_4_I_4_/TiO_2_ and b) used Cu_4_I_4_/TiO_2_.

**Table S6.** The copper ion concentration determined by ICP-OES before and after the photocatalytic nitrate reduction reaction.

| Sample | Copper ion concentration (mg/L) |
| --- | --- |
| Before illumination | 0.21 |
| After illumination | 0.19 |

**Figure S30.** Py-IR spectra of TiO_2_ and Cu_4_I_4_/TiO_2_ after the reaction.

**Figure S31.** a) Cu 2p, b) I 3d, and c) Ti 2p XPS spectra of the used catalysts.

**Figure S32.** TGA curve of Cu_2_I_2_Py_4_.

**Figure S33.** The crystal structure of a) Cu_2_I_2_Py_4_ nanocluster and b) Cu_2_I_2_ core.

**Figure S34.** a) NH_4_^+^ production for a series of Cu based catalysts. b) Selectivity and conversion tests for a series of Cu based catalysts.

**Table S7** NH_3_-TPD characterization results of catalysts.

| NH_3_ desorption amount (μmol/g) | |
| --- | --- |
| Catalyst | Total |
| TiO_2_ | 66.7 |
| Cu_4_I_4_/TiO_2_ | 116.18 |

**Table S8** Distribution of Brønsted and Lewis acids on different samples.

| Catalyst | Brønsted  (integrated area) | Lewis  (integrated area) | Total  (integrated area) | B/L |
| --- | --- | --- | --- | --- |
| TiO_2_ | 0.062 | 0.417 | 0.479 | 0.148 |
| Cu SA/TiO_2_ | 0.046 | 0.213 | 0.259 | 0.217 |
| CuI NP/TiO_2_ | 0.041 | 0.389 | 0.430 | 0.106 |
| Cu_4_I_4_/TiO_2_ | 0.042 | 0.728 | 0.770 | 0.057 |

**Figure S35.** Py-IR spectra of Cu SA/TiO_2_ and CuI NP/TiO_2_.

**Figure S36.** a) EIS Nyquist plots of TiO_2_ and Cu_4_I_4_/TiO_2_. b) Transient photocurrent responses for the TiO_2_ and Cu_4_I_4_/TiO_2_. c) Time-resolved photoluminescence (TRPL) profiles of TiO_2_ and Cu_4_I_4_/TiO_2_ under ambient conditions (λ_ex_ = 375 nm).

**Figure S37.** In situ EPR investigation toward TEMPO concentration consumption for DI oxidative half reaction and HCOOH oxidative half reaction over TiO_2_.

**Figure S38.** In situ Ti 2p XPS spectra of Cu_4_I_4_/TiO_2_ under varied irradiation conditions.

**Figure S39.** In situ ATR-FTIR spectra of the adsorption peaks of NO_3_^−^ reduction over Cu_4_I_4_/TiO_2_.

**Figure S40.** In situ ATR-FTIR spectra of the adsorption peaks of HCOOH oxidation over Cu_4_I_4_/TiO_2_.

**Figure S41.** In situ ATR-FTIR spectra of the adsorption peaks of NO_3_^−^ reduction and HCOOH oxidation over a) TiO_2_ and b) Cu_4_I_4_/TiO_2_.

**Figure S42.** In situ ATR-FTIR spectra of the adsorption peaks of NO_3_^−^ reduction over a) TiO_2_, b) Cu SA/TiO_2_ and c) Cu_2_I_2_/TiO_2_. In situ ATR-FTIR spectra of the adsorption peaks of HCOOH oxidation and NO_3_^−^ reduction over d) TiO_2_, e) Cu SA/TiO_2_ and f) Cu_2_I_2_/TiO_2_.

**Figure S43.** Side views of charge density difference for Cu_4_I_4_/TiO_2_, Cu_2_I_2_/TiO_2_, and TiO_2_ with the adsorbed nitrate. The cyan and yellow iso-surfaces (iso-value = 0.03 electrons/Å^3^) represent charge depletion and accumulation, respectively.

**Figure S44.** Charge transfers between Cu_4_I_4_/TiO_2_, Cu_2_I_2_/TiO_2_, and TiO_2_ with the adsorbed nitrate.

**Figure S45.** PDOS of TiO_2_ a) before and b) after nitrate adsorption.

**Figure S46.** Adsorption energies of the water on the TiO_2_, Cu SA/TiO_2_, and Cu_4_I_4_/TiO_2_.

**Figure S47.** Side views of the structure for the reduction of the nitrate to ammonia on the Cu_4_I_4_/TiO_2_ through primary reaction.

**Figure S48.** Side views of the structure for the reduction of the nitrate to ammonia on the Cu_4_I_4_/TiO_2_ through secondary reaction.

**Figure S49.** Side views of the structure for the reduction of the nitrate to ammonia on the TiO_2_.
